# Emerging socioeconomic correlates of loneliness. Evidence from the Barcelona Health Survey 2021

**DOI:** 10.1007/s00127-024-02789-w

**Published:** 2024-11-18

**Authors:** Lluís Mangot-Sala, Xavier Bartoll-Roca, Esther Sánchez-Ledesma, Mònica Cortés-Albaladejo, Aart C. Liefbroer, Katherine Pérez

**Affiliations:** 1 Netherlands Interdisciplinary Demographic Institute (NIDI) – Royal Netherlands Academy of Sciences (KNAW), The Hague, The Netherlands; 2 Department of Epidemiology, University Medical Center Groningen (UMCG), University of Groningen (RUG), Groningen, The Netherlands; 3 Agència de Salut Pública de Barcelona (ASPB), Barcelona, Spain; 4 Unit of Occupational Medicine, Institute of Environmental Medicine, Karolinska Institutet, Stockholm, Sweden; 5 Institut d’Investigació Biomèdica Sant Pau (IIB SANT PAU), Barcelona, Spain; 6 CIBER Epidemiología y Salud Pública (CIBERESP), Madrid, Spain; 7 Department of Sociology, Vrije Universiteit Amsterdam (VU), Amsterdam, The Netherlands

**Keywords:** Precarity, Material deprivation, Loneliness, Housing conditions, Financial insecurity, Correlates of loneliness

## Abstract

**Purpose:**

Recent evidence shows that loneliness is associated with socioeconomic factors. However, studies often focus on traditional socioeconomic indicators (income, occupation, educational level) only, disregarding other important socioeconomic determinants, such as job insecurity, housing conditions or material deprivation. Therefore, we analyse the association of a broad range of socioeconomic indicators with loneliness. Moreover, we investigate potential age and gender differences in this relationship.

**Methods:**

We used cross-sectional data from the Barcelona Health Survey 2021, representative of the population of Barcelona (Spain). Individuals over the age of 14 were selected (*n* = 3,337). The outcome was a loneliness score based on 4 items of the UCLA scale. Loneliness was regressed on a series of sociodemographic and emerging socioeconomic correlates. Linear regression models were fitted, and potential age and gender moderation effects were tested by means of two-way interactions.

**Results:**

Job insecurity and precarity-related factors, such as having a temporary job or working without a contract, material deprivation and financial difficulties, as well as poor housing conditions and facing housing insecurity were associated with increased loneliness levels. While the association between loneliness and precarity-related factors is stronger among younger individuals, material deprivation is associated with increased loneliness among older workers and women.

**Conclusion:**

Beyond sociodemographic individual characteristics, socioeconomic factors are strongly associated with loneliness levels in the population. Findings support the relevance of broadening the scope of socioeconomic indicators, assessing both material conditions as well as perceived insecurity.

**Supplementary Information:**

The online version contains supplementary material available at 10.1007/s00127-024-02789-w.

## Introduction

Loneliness is usually defined as a discrepancy between the desired and the actual quality and quantity of social connections an individual has [1, 2]. Loneliness is an increasing concern in Western societies [3], and is known to have numerous negative consequences in terms of health and well-being [1]. Evidence shows that individuals who feel lonely are at a higher risk of early all-cause mortality [4], cardiovascular disease [5], mental health issues [6], and alcohol and substance use disorders [7].

Traditionally, research has focused on older individuals, considered to be particularly vulnerable to loneliness [8, 9]. However, recent studies suggest that nowadays younger individuals report the highest loneliness levels [10], this being particularly true during the Covid-19 pandemic [11, 12]. A potential explanation is that younger people tend to have larger social networks and rely more strongly on them than older individuals [13]. For that reason, they particularly suffered the consequences of the drastic restrictions in social contacts imposed by the pandemic [12].

Although loneliness was traditionally analysed mainly as a risk factor for mental and physical health outcomes [7, 9, 11, 14], in the last decades, numerous studies have aimed to identify the determinants of loneliness itself. Most of these studies focused on sociodemographic factors (i.e. gender, partner/marital status, living arrangement [15]), as well as social network and social engagement [8, 9]. Yet, the role of socioeconomic factors was largely ignored. In fact, until recent years, loneliness was considered “the great equalizer” [11], as it was supposed to affect large parts of the population, regardless of gender, socioeconomic status [16] or ethnicity [1].

However, recent studies that incorporated socioeconomic determinants partly contradicted these findings [17], showing that loneliness is related to people’s socioeconomic situation, such as educational level, employment, or income, net of demographic and psychological factors [18, 19]. For instance, evidence shows that individuals with lower education are more likely to feel lonely [18], allegedly because higher education may offer more, and better, opportunities for social interaction, as well as better relational skills [14]. Similarly, loneliness seems to be more prevalent among those who experience financial deprivation [10, 20], allegedly because financial resources increase the participation in social activities, and, therefore, a lack thereof may prevent building solid networks [14].

Recently, some authors have argued that the scope of socioeconomic indicators should be broadened, stressing the role of “emerging” socioeconomic determinants, such as job insecurity, housing accessibility, as well as “precarious living conditions” in general [3, 15, 17]. Such socioeconomic factors may be relevant for two main reasons: first, the traditional socioeconomic status (SES) indicators -educational level, income, occupation- are known to have some limitations [21]. For instance, educational level may not be a good proxy in saturated labour markets [22], where higher education does not necessarily lead to highly qualified jobs. In turn, income is often underreported [21, 23], and occupation can fail to capture extremely different working conditions within the same occupational categories^1^, not to mention that not everyone works [23]. Second, while material resources may be relevant, recent evidence shows that socioeconomic *prospects* -i.e. concern over a potential lack of resources in the future- may be significantly related to loneliness as well [17]. This study incorporates both traditional and emerging socioeconomic indicators, including socioeconomic prospects.

It is well known that precarious employment and perceived job insecurity -i.e. the perceived risk of losing one’s job in the near future- affect large parts of the population and seem to be on the rise [24]. In the Spanish context, this is particularly true for young people [25]. Furthermore, housing has become increasingly unaffordable, which implies that both precarious housing conditions and housing insecurity -i.e. being forced to move in the near future- have become relatively widespread in Barcelona -a city with less than 2% of public housing [26]-, and other Southern European cities [27]. Since both precarious employment and housing are known to strongly affect mental health [26], it is reasonable to think that they may affect loneliness levels as well.

Moreover, there are reasons to believe that the role of such “emerging socio-economic factors” may have been particularly significant during the COVID-19 pandemic. First, financial or job insecurity may have contributed to the overall high levels of fear and uncertainty raised by the pandemic [28]. Second, it is known that precarious working conditions contribute to isolate individuals, as they hamper building meaningful connections, e.g. with colleagues or customers [29]. That may be particularly relevant during the pandemic, as social interactions were drastically reduced, and social support -which may otherwise have worked as a stress-relief- was reduced to minimum levels or, at best, turned into virtual contact, which has been shown to be often counter-effective [30], even increasing loneliness in some cases.

On the other hand, evidence suggests that the role of socioeconomic determinants of loneliness may differ across subgroups of individuals, depending on individual characteristics such a age or gender [14]. Matthews et al. (2019) showed that loneliness was mainly affected by socioeconomic determinants in older adults, whereas for younger people loneliness was unrelated to socioeconomic characteristics [10]. De Jong Gierveld reached a similar conclusion back in 1998, when she showed that, among young people, loneliness was mainly related to personality traits [31]. Nevertheless, recent studies conducted during the COVID-19 pandemic provided slightly different insights: not only did younger individuals report the highest loneliness levels during the lockdown periods [3, 12], but employment-related variables, such as job insecurity, appeared as particularly consequential [3, 17] for younger groups. A potential explanation is that such insecurity may increase uncertainty in the partner and family domain, as recently shown [32]. Moreover, it can hamper young people’s ability to commit to relationships, such as partnership or long-lasting friendship, which are known risk factors for loneliness [33]. However, evidence is scarce and restricted to a limited number of socioeconomic indicators.

In turn, studies consistently showed higher loneliness levels among women [34], and a stronger increase in their loneliness levels during the COVID-19 pandemic [35], although evidence regarding gender differences in the role of socioeconomic determinants of loneliness is scarce. The few existing studies showed a stronger association between material deprivation during early life and loneliness levels later in life among women [33, 36], allegedly because, due to socialization differences and gender roles, low social status was a stronger impediment to present women as “attractive partners” than it was for men [36], which could undermine their self-worth, and hamper the connection with others. However, no study has specifically analysed gender differences regarding emerging socioeconomic determinants of loneliness. It could be argued that job insecurity and precarious living conditions often hinder achieving satisfactory work-life balance, which may be more consequential for women, since they carry heavier burden of care and family work [37].

In sum, while some studies have analysed socioeconomic determinants of loneliness, most rely on traditional SES indicators, which may be insufficient in the current context, particularly among young people. Despite the recommendations to use additional SES assessments [3, 17], such as housing conditions, job insecurity or experiences of deprivation, most studies lack the data to do so. To the best of our knowledge, this is the first study to analyse a large set of socioeconomic correlates of loneliness, assessing both material conditions and prospects. Furthermore, we examine whether the association between SES and loneliness differs by age group and gender.

The main aims of this study are:

1. *To analyse whether employment-related*, *housing-related and financial-related indicators were associated with loneliness.*
2. *To analyse potential age and gender differences in the role of these correlates.*

## Materials and methods

### Data and study population

We used data from the Barcelona Health Survey 2021 (BHS). The BHS is a cross-sectional health survey based on a representative sample (N≃4,000) of the non-institutionalized population. To ensure territorial representativeness, the sample was stratified by municipal district. A random sample was drawn from the municipal census, considering gender and age distribution. Data was collected by computer-assisted face-to-face interviewing [38]. The BHS is particularly rich on information on health and socioeconomic determinants of health, which makes it appropriate to analyse their role as correlates of loneliness. For this study, individuals younger than 14 years old (*n* = 412) and those unable to respond to the survey (*n* = 219) were excluded, which resulted in final sample of *n* = 3,337 individuals. Sampling weights by gender, age, and city district were applied to ensure representativeness of the sample.

### Measurements

#### Outcome

Self-reported loneliness was assessed using four items from the UCLA Loneliness Scale (“How often do you feel that you lack companionship?”, “How often do you feel left out?”, “How often do you feel isolated from others?”, and “How often do you feel alone?”). Items were rated *never*/*hardly ever* (0), *sometimes* (1), or *often/very often* (2) and were summed resulting in a loneliness score ranging from 0 to 8, as previous studies did [10, 39]. The four items showed good internal consistency (Cronbach’s Alpha = 0.84).

#### Sociodemographic determinants

Age was categorized in four groups (< 35 years, 35–50, 51–65, > 65), as in the Youth Survey of Barcelona for comparative purposes [40]; *Gender* (men/women); *Living arrangement* (Lives alone, cohabiting with others but not a partner, cohabiting with partner but unmarried, and married); and *Migration status* (“Spanish”, and “Other nationality”).

#### Socioeconomic determinants

The following set of socioeconomic variables were assessed:

Educational level (Primary education or lower, secondary education, university/higher education).

#### Employment-related variables

(A) Employment status (Employed, retired, homemaker, unemployed, disabled, student/other). (B) Contract situation was assessed via dummy variables: “Part-time job”, “Temporary contract”, “Working as a freelance” and “Working without a contract” (yes/no). (C) Job insecurity was assessed via the question: “How likely it is to lose your job in the coming 3 months?”, an indicator used in the European Working Conditions Survey [41]. Those who answered “likely” or “very likely” were considered to experience job insecurity; (D) Employment during covid-19 compared those “employed as usual” (reference category), with those “temporarily furloughed”^2^, or “laid-off” due to the pandemic.

#### Housing-related variables

A) Housing conditions were assessed based on the following items: “Having leaks/humidities”, “Having insects/mice”, and “Having fumes/pollution from outside” in the house [42]. The scores were summed into a categorized variable: “Good” (0 items), “Fair” (1 item) and “Poor” housing conditions (2–3 items). B) Housing insecurity was assessed via the question: “Are you forced to move out of your current house within the next 6 months?”. Those who answered positively were considered to experience housing insecurity. C) House tenancy status was assessed, comparing homeowners (ref.), with those who have a mortgage, tenants, and those in “other” situations (e.g. living with family members).

#### Financial situation

(A) Material deprivation was defined as “not being able to afford three or more of the following items: washing machine, TV, computer, car, phone, internet connection, one week annual holidays away from home, meat/fish once a week, unexpected expenses of 750€, to pay for arrears (mortgage/rent, utility bills) and not being able to keep the dwelling warm” [42]. (B) Financial insecurity was assessed via the question: “How do you “make ends meet” at the end of the month?” Score 1–6 (1 = Very comfortably; 6 = With great difficulty). Categorized into “No/little difficulties” (score ≤ 2), “Moderate difficulties” (score 3–4), and “Great difficulties” (score ≥ 5) [42]^3^.

Additionally, given that loneliness is strongly related with *mental health status*, we accounted for it as sensitivity analyses, to test whether the association between socioeconomic indicators and loneliness remained after accounting for mental health. They were measured using the General Health Questionnaire (GHQ-12), a screening test proved to be a reliable instrument to detect people at risk poor mental health consisting of 12 items (range 0–12), with a cut-off point of ≥ 3 [43] (the 12 items are shown in the Supplementary File).

### Statistical analyses

First, univariate descriptive analyses of the main variables of interest were performed. Next, self-reported loneliness levels across these variables were compared by means of Anova and t-tests. Second, ordinary least squares (OLS) regression models were performed, regressing loneliness scores on sociodemographic and socioeconomic covariates in a stepwise fashion. Determinants were considered significant at p-values lower than 0.05. Potential collinearity between the different determinants was tested with the “variance inflation factor” (VIF) test. Missing values were imputed using multiple chained equation models (MICE), resulting in practically identical results (Table A1 in the Supplementary File). Hence, we present the models with observed values to facilitate further postestimation tests. Third, potential gender and age differences were assessed by adding interaction terms to the main effects model and stratifying by gender (Tables A2.1 & A2.2 in the Supplementary File). Predicted values for the different categories were estimated via margins. Last, a model containing only the subsample of individuals reporting good mental health (Table A3 in the Supplementary File) was fitted to test the potential mediating role of mental health. All analyses were conducted using Stata 14.0.

## Results

The main characteristics of the study sample (*n* = 3,337) are described in Table 1. Mean loneliness scores, broken down by the main variables of interest, are presented. Most participants were women (53%) and the average age was 49 years. Average loneliness score in the sample was 0.94, with significant variation between groups: individuals under 35 years old reported a higher loneliness score (1.07) than their older peers, whereas differences between the other age groups are negligible (0.89 for those between 35 and 50 and those > 65; 0.91 for those between 51 and 65 years). Women reported higher loneliness than men (1.06 by 0.80 respectively). Individuals living alone reported the highest loneliness levels (1.61), followed by those cohabiting with others, but not a partner (1.20), those cohabiting with a partner but unmarried (0.99) and married individuals (0.63) Individuals with lower education reported higher loneliness (1.12 by 0.79 among the highly educated), whereas those with a Spanish nationality were less lonely (1.20) than those with other nationalities (0.89). In turn, unemployed individuals (1.30), and particularly those with an occupational disability (1.93) showed significantly higher prevalence of loneliness than retired individuals (0.84) or full-time employed (0.77).

Loneliness levels strongly differed by socioeconomic factors. Among employed individuals, those who experienced job insecurity were significantly lonelier (1.71), and so were, those who were laid-off due to the Covid-19 pandemic, who scored higher than those who kept their jobs (1.18) or were only temporarily furloughed (0.89). Those having a temporary contract (1.24) or working without contract (1.51) also reported higher loneliness levels than peers with a permanent contract. In contrast, freelancers reported slightly lower prevalence of loneliness (0.72) than the rest of employed individuals.

Those facing housing insecurity (1.75) or poor housing conditions also showed higher loneliness than their peers with proper and stable housing. Moreover, the latter showed a clear gradient, with those reporting “poor” conditions having higher loneliness levels (1.55) than those with “fair” conditions (1.25), which in turn were higher than among those with “good” conditions (0.83). Tenants reported slightly higher loneliness (0.99) than their peers, although differences were small (whereas differences between those with a mortgage and those who owned the house were negligible). Last, experiencing material poverty (1.62), or strong financial difficulties (1.74) was also associated with higher loneliness levels, the latter also showing a clear dose-response pattern.

**Table 1:** Univariate and bivariate descriptive analyses of the main variables of interest. Sample > 14 years (*n* = 3,337)

				UCLA 4-item score (0–8)
	*n* (%)	Missing (%)	Mean score (SD)	*p*
*Age (mean; SD) || UCLA4 (mean; SD)*	*48.97 (18.89)*	*0 (0.00%)*	*0.94 (1.69)*	
Age group		*0 (0.00%)*		*0.08*
< 35	875 (26.07%)		1.07 (1.71)	
35–50	980 (29.37%)		0.89 (1.65)	
51–65	753 (22.57%)		0.91 (1.73)	
> 65	734 (22.00%)		0.89 (1.66)	
Gender		*9 (0.27%)*		0.00**
Men	1,557 (46.66%)		0.80 (1.59)	
Women	1,771 (53.07%)		1.06 (1.75)	
Living arrangement		*9 (0.27%)*		0.00**
Alone	387 (11.60%)		1.61 (2.06)	
Cohab. with others (not partner)	649 (19.45%)		1.20 (1.89)	
Cohab. with partner (not married)	809 (24.24%)		0.99 (1.62)	
Cohabiting married	1,483 (44.44%)		0.63 (1.44)	
Educational Level		*7 (0.21%)*		0.00**
Primary	1,039 (31.14%)		1.12 (1.92)	
Secondary	917 (27.48%)		0.97 (1.72)	
University	1,374 (41.17%)		0.79 (1.45)	
Nationality				0.00**
Spanish	2,810 (84.21%)	*1 (0.03%)*	0.89 (1.65)	
Other	526 (15.76%)		1.20 (1.85)	
Employment status		*26 (0.78%)*		0.00**
Employed (full-time)	1,531 (45.88%)		0.77 (1.47)	
Employed (part-time)	352 (10.55%)		1.13 (1.87)	
Retired	682 (20.44%)		0.84 (1.63)	
Unemployed	258 (7.73%)		1.30 (1.94)	
Homemaker	171 (5.12%)		1.24 (1.89)	
Disability	90 (2.70%)		1.93 (2.56)	
Student/Other	227 (0.78%)		1.12 (1.76)	
Work during Covid		*3 (0.09%)*		0.00**
Employed as usual	1,370 (41.05%)		0.79 (1.51)	
Furloughed (temporarily)	386 (11.57%)		0.89 (1.67)	
Lost job (laid-off)	124 (3.72%)		1.18 (1.76)	
N/A^a^	1,454 (43.57%)			
Job Insecurity		*53 (1.59%)*		0.00**
No insecurity	1,684 (50.46%)		0.75 (1.44)	
Job Insecurity	146 (4.38%)		1.71 (2.22)	
N/A	1,454 (43.57%)			
Temporary job		*6 (0.18%)*		0.00**
No	1,630 (48.85%)		0.78 (1.52)	
Yes	247 (7.40%)		1.24 (1.79)	
N/A	1,454 (43.57%)			
Freelance		*6 (0.18%)*		0.00**
No	1,670 (50.04%)		0.85 (1.58)	
Yes	207 (6.20%)		0.72 (1.44)	
N/A	1,454 (43.57%)			
Work without contract		*6 (0.18%)*		0.00**
No	1,826 (54.72%)		0.82 (1.53)	
Yes	51 (1.53%)		1.51 (2.44)	
N/A	1,454 (43.57%)			
Housing Conditions		*0 (0.00%)*		0.00**
Good	2,555 (76.57%)		0.83 (1.58)	
Fair	613 (18.37%)		1.25 (1.95)	
Poor	169 (5.06%)		1.55 (1.97)	
Housing Tenancy Status		*37 (1.11%)*		0.00**
Owner (paid)	1,276 (38.24%)		0.86 (1.64)	
Owner (mortgage)	692 (20.74%)		0.89 (1.58)	
Tenant	1,106 (33.14%)		0.99 (1.69)	
Other	226 (6.77%)		1.26 (2.13)	
Housing Insecurity		*67 (2.01%)*		0.00**
No insecurity	3,150 (94.40%		0.90 (1.64)	
Insecurity	120 (3.60%)		1.75 (2.44)	
Material Deprivation		*15 (0.45%)*		0.00**
No deprivation	2,759 (82.68%)		0.81 (1.52)	
Deprivation	563 (16.87%)		1.62 (2.26)	
Financial difficulties		*59 (1.77%)*		0.00**
No/little difficulties	1,146 (34.34%)		0.69 (1.41)	
Moderate difficulties	1,676 (50.22%)		0.90 (1.60)	
Strong difficulties	456 (13.66%)		1.74 (2.34)	

*p-value < 0.05; **p-value < 0.01; ^a^ Not applicable = the respondent is not employed

### Correlates of loneliness

Table 2 shows the estimates from the regression analysis. None of the covariates showed significant collinearity levels (all VIF values were < 2, except for “retired” and “>65 years old”, with VIF≃4). As shown in the main effects model (Model 1), individuals over 65 were somewhat less likely to feel lonely (b=-0.25) than their younger peers (those under 35), although differences are not statistically significant. In turn, living arrangement appeared strongly associated with the outcome: compared with married individuals, those living alone had significantly higher loneliness levels (b = 1.01), followed by those living with others, but not a partner (b = 0.43), and those cohabiting unmarried with a partner (b = 0.39). Those with a foreign nationality were also more likely to feel lonely than Spanish nationals (b = 0.29).

Turning to socioeconomic variables, some employment-related factors were associated with loneliness: loneliness was significantly higher among homemakers (b = 0.63), retired individuals (b = 0.36) and individuals with occupational disabilities (b = 1.06), who showed the highest loneliness levels of the whole sample. Moreover, job insecurity was a significant risk factor for loneliness (b = 0.49), whereas working without a contract (b = 0.36) and having a temporary job (b = 0.19) showed a weaker, non-statistically significant, association. In turn, the specific employment situation experienced during the Covid-19 pandemic (i.e. being furloughed or laid-off) did not appear significantly associated with loneliness levels.

Second, while housing tenancy status did not seem associated with loneliness levels, housing conditions showed a dose-response pattern with the outcome. Thus, compared with those who report having “good” housing conditions, those with suboptimal (“fair”) housing conditions had higher loneliness levels (b = 0.26), which increased even more among those with “poor” housing conditions (b = 0.46), i.e. those experiencing leaks, insects and/or pollutants in the house. In turn, experiencing housing insecurity (i.e. being forced to move soon) was also associated with increased loneliness levels (b = 0.45).

Last, material conditions were associated with loneliness levels, as shown by the significant effect of experiencing material deprivation on loneliness levels (b = 0.31). Yet, the association was much stronger for severe financial difficulties: individuals reporting having great difficulties to “make ends meet” at the end of the month had significantly higher loneliness levels (b = 0.65), net of other sociodemographic and housing- or employment-related factors.

**Table 2:** Determinants of loneliness. OLS regression models

	Model 1 (*n* = 3,078)	
	b	95% CI
Gender (Men)		
Women	0.09	(-0.02; 0.21)
Age (< 35)		
35–50	0.01	(-0.16; 0.18)
51–65	0.02	(-0.18; 0.22)
> 65	-0.25	(-0.55; 0.06)
Living arrangement (Married)		
Alone	1.01**	(0.81; 1.20)
Cohabit. with others (not partner)	0.43**	(0.26; 0.59)
With partner	0.39**	(0.22; 0.56)
Educational Level (University)		
Primary (or lower)	-0.01	(-0.16; 0.14)
Secondary	-0.04	(-0.19; 0.10)
Nationality (Spanish)		
Other	0.29**	(0.12; 0.47)
Employment status (Employed full-time)		
Employed part-time	0.18	(-0.02; 0.38)
Unemployed	0.30*	(0.06; 0.53)
Homemaker	0.63**	(0.31; 0.94)
Retired	0.36*	(0.08; 0.63)
Disabled	1.06**	(0.68; 1.44)
Work during Covid (Employed as usual)		
ERTO (Furloughed temporarily)	0.01	(-0.19; 0.19)
Lost job (laid-off)	0.00	(-0.32; 0.32)
Job Insecurity (No insecurity)		
(High) Insecurity	0.49**	(0.20; 0.78)
Temporary job (No)		
Yes	0.19	(-0.05; 0.43)
Freelance (No)		
Yes	-0.03	(-0.28; 0.21)
Work without contract (No)		
Yes	0.36	(-0.12; 0.84)
House tenancy (Owner; paid)		
Mortgage	-0.03	(-0.20; 0.13)
Tenant	-0.13	(-0.29; 0.02)
Housing Conditions (Good)		
Fair	0.26**	(0.11; 0.40)
Poor	0.46**	(0.19; 0.73)
Housing Insecurity (No insecurity)		
Insecurity	0.45**	(0.13; 0.77)
Material Poverty (No)		
Poverty	0.31**	(0.12; 0.48)
Financial difficulties (No difficulties)		
Moderate difficulties	0.10	(-0.02; 0.23)
Great difficulties	0.65**	(0.44; 0.87)

*p-value < 0.05; **p-value < 0.01

### Gender and age differences

**Fig. 1:**
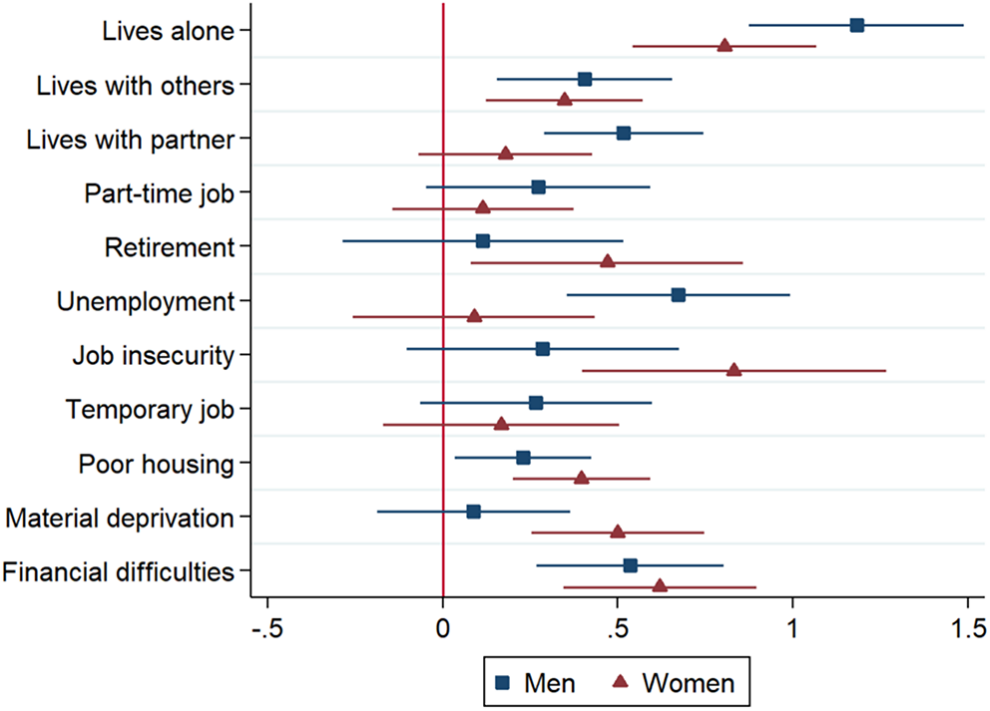
Correlates of loneliness by gender. Predicted margins based on stratified OLS regression models

First, differences between men and women were tested by adding to the main effects model an interaction term between gender and the different socioeconomic correlates. As shown by the significant interaction terms presented in Table 3 (Model 2), gender significantly moderated the association between material deprivation and loneliness, and between job insecurity and loneliness, whereas interactions with the other socioeconomic correlates (not shown) were not statistically significant. Thus, both job insecurity as well as material deprivation appear more consequential for women than for men, as shown by both strongly significant interaction terms (b = 0.58 and b = 0.45 respectively). Additionally, to better grasp gender differences, we also stratified the main model (see tables A2.1 and A2.2 in the Supplementary File). Figure 1 shows the coefficients of the model stratified by gender.

**Table 3:** Material deprivation and job insecurity as determinants of loneliness. Interaction^a^ with gender (model 2) and age group (model 3)

	Model 2 (*n* = 3,078)		Model 3 (*n* = 3,078)	
	b	95% CI	b	95% CI
Job Insecurity (No insecurity)				
(High) Insecurity	0.23	(-0.17; 0.63)	1.00**	(0.54; 1.46)
Job Insecurity * Age				
(High) Insecurity * 36–50 years			-0.89**	*(-1.53; -0.25)*
(High) Insecurity * 51–65 years			*-0.61*	*(-1.38; 0.15)*
(High) Insecurity * >65 years			*-1.07*	*(-5.48; 3.34)*
*Job Insecurity ## Gender*				
High insecurity * Women	0.58*	*(0.01; 1.14)*		
Material Deprivation (No)				
Deprivation	0.03	(-0.24; 0.30)	-0.23	(-0.55; 0.09)
Deprivation * Age				
Deprivation * 36–50 years			0.52*	*(0.10; 0.93)*
Deprivation * 51–65 years			1.30**	*(0.83; 1.77)*
Deprivation * >65 years			0.51*	*(0.05; 0.96)*
Deprivation * Gender				
Deprivation * Women	0.45**	*(0.13; 0.77)*		

*p-value < 0.05; **p-value < 0.01

^a^ Based on the main effects model (Table 2; Model 1)

**Fig. 2:**
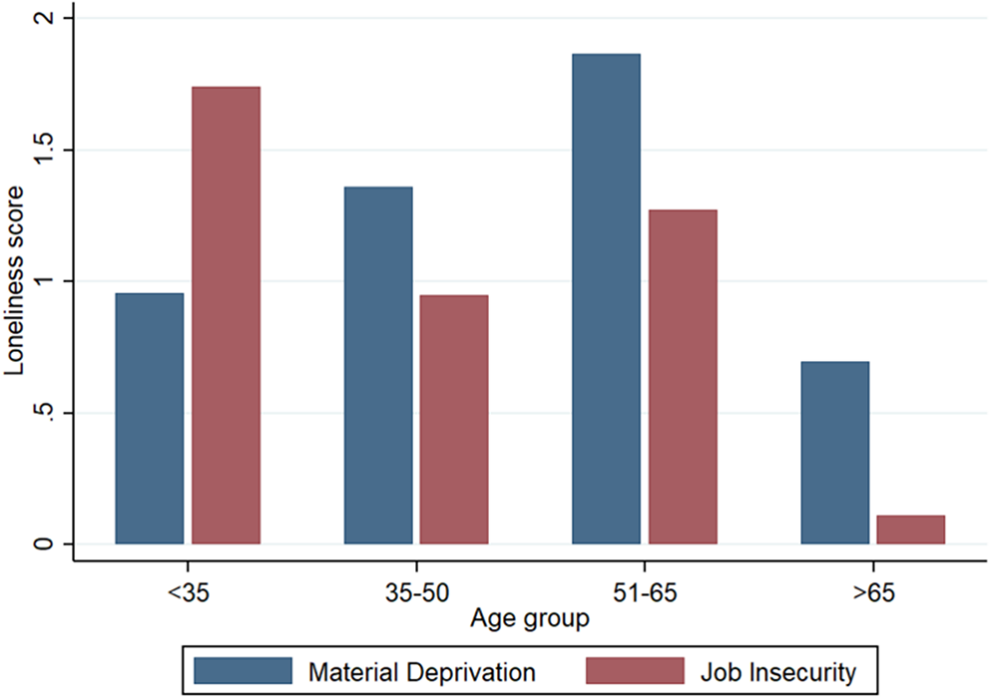
Age differences in the association between Material Deprivation and Job insecurity and Loneliness

Second, the role of age as moderator was assessed by adding an interaction term between age and socioeconomic determinants to the main effects model. As shown in Model 3 of Table 3, age significantly moderated the association between job insecurity and loneliness. While job insecurity strongly affected loneliness levels among younger individuals (b = 1.00 for those < 35), a strong negative significant interaction term (b=-0.89) among those 36–50 years, suggests that job insecurity was significantly less consequential for this age group. Please note that, since it is an employment-related indicator, it is not relevant for individuals in retirement age (hence the large confidence interval in the oldest age group). Moreover, age also moderated the association between material deprivation and loneliness. While, in this case, the coefficient was not significant for the reference category (i.e. b=-0.23 for those < 35), the effect of material deprivation on loneliness showed a significant increase with age: material deprivation was significantly more consequential for those 36–50 (interaction coefficient b = 0.52), and even more for those 51–65 years (b = 1.30), who reported the strongest association between material deprivation and loneliness. These interactions are represented in Fig. 2, which shows the estimated loneliness levels depending on job insecurity and material deprivation, by age group.

In sum, results show that, beyond sociodemographic elements such as living arrangement (living alone was a risk factor, especially for men), socioeconomic indicators were significant correlates of loneliness. Thus, job insecurity, and other precarity-related factors, such as having a temporary job, or working without a contract were associated with increased loneliness levels. Similarly, material deprivation, such as financial difficulties or poor housing conditions, was also associated with increased loneliness. These associations appeared somewhat stronger for women and showed a clear age pattern: while precarity-related variables were particularly consequential for younger individuals, material deprivation and financial difficulties (to a lesser degree housing insecurity as well) were especially significant among older workers.

## Discussion

This study, based on a representative sample of the population of Barcelona, shows that loneliness levels were strongly associated with socioeconomic factors. Its main contribution is to incorporate a broad range of indicators related to different socioeconomic domains, such as employment, housing, and the financial situation of the participants. Moreover, both current material conditions, as well as anticipated economic prospects, such as perceived job or housing insecurity were assessed. Results showed that job insecurity, and other precarity-related elements, such as having a temporary job or no employment contract, were associated with higher loneliness levels. Likewise, having poor housing conditions, housing insecurity, and material deprivation were significant correlates of loneliness as well. Moreover, the associations between socioeconomic factors and loneliness appeared particularly strong for women, and results differed by age as well: while job insecurity was particularly associated with higher loneliness levels among younger individuals, material deprivation particularly affected older workers.

By assessing the role of socioeconomic correlates, our study overcomes some of the limitations of studies, namely: focusing only on sociodemographic characteristics or social network size [31, 44]. Furthermore, our study focuses on *emerging* socioeconomic determinants, i.e. socioeconomic factors that were identified as particularly relevant determinants of health in the last decades [15], such as poor or precarious housing conditions and job insecurity. Thus, our study overcomes some of the limitations of traditional SES assessments [8, 14, 18, 20]. This is particularly relevant in the Southern-European context, where a succession of severe economic crises has deeply transformed the traditional correlation between education, occupation, and income [25], and precarious housing and employment conditions have become widespread [27].

First, results showed that experiencing material deprivation and financial difficulties was associated with increased loneliness levels. This finding aligns with previous studies reporting an association between loneliness levels and living conditions, such as employment situation [18] or financial hardship [45], allegedly because a lack of material resources often hampers social life and may lead to social isolation [14]. Furthermore, our study adds evidence regarding the role of housing conditions, a largely unexplored dimension of material deprivation. Although a few studies had incorporated housing-related assessments to their analyses, mainly tenancy status [46] or housing type [3], to the best of our knowledge, our study is the first to show a dose-response pattern between housing deficiencies and loneliness levels, accounting for other socioeconomic indicators. Although poor mental health may certainly play a key role in the association between housing deficiencies and loneliness, sensitivity analyses revealed that the association was still significant after accounting for mental health, suggesting other mechanisms, e.g. increased social exclusion levels [17].

Moreover, housing insecurity -i.e. the concern about being forced to move in the near future- also appeared as a risk factor of loneliness. That aligns with recent findings suggesting that having *concerns* over a potential lack of resources in the future (including housing) predicted loneliness levels more than the actual material living conditions [17]. Consistently, our findings showed that indicators related with poor economic *prospects*, such as precarious employment conditions were associated with increased loneliness levels, net of current material conditions. Although literature on the association between precarity and loneliness is still scarce, potential explanations have been proposed: on a psychosocial level, precarious employment conditions are associated with poor mental health [47, 48] and lower self-efficacy levels [49], which may hamper social connectivity, potentially increasing loneliness levels [20, 34]. On a structural level, precarity increases the risk of social exclusion [17], which in turn is strongly associated with loneliness [50]. Anyhow, further research is needed to disentangle the pathways leading from precarious housing or working conditions to higher loneliness levels.

Second, although loneliness was believed to mainly affect older individuals, our findings coalesce with recent studies consistently showing that younger people report the highest loneliness levels [7, 10], a trend that became particularly evident during the pandemic [11, 12]. Furthermore, our study shows that loneliness among younger individuals was related with precarious employment conditions. This finding has several implications. First, it contradicts previous evidence suggesting that loneliness among younger individuals is unrelated to socioeconomic factors [16], and mainly driven by specific personality traits [31]. Second, precarity could partly explain the high loneliness levels reported by young people, as they may perceive employment insecurity as the main obstacle preventing them from, for instance, accessing decent housing or forming a family [24]. Third, our findings stress the need for new indicators to capture the current context of employment [48], as the significant role of job insecurity as a determinant of loneliness would have been missed had we relied only on traditional SES indicators.

Third, results showed that the association between loneliness and both job insecurity and material deprivation was stronger for women. Recent studies had shown higher loneliness levels among women [34], especially during the COVID-19 pandemic [3], allegedly because women tend to depend more on social connections than men [3], and therefore suffered more the lack of social contacts caused by the lockdown measures [12]. However, to the best our knowledge, our study is the first to show a stronger role of socioeconomic factors for women. The fact that women face higher precarity in the labour market [51], and suffer more material deprivation [52], may help explain these findings. Moreover, women also provide more informal and family care than men, which may also suppose greater strain on them, especially in times of increased family interactions during the lockdown [53].

Last, our findings showed that individuals with occupational disabilities, homemakers and, to a lesser degree, retired individuals feel lonelier than their employed peers, arguably because these groups may share a feeling of being “excluded” from the labour market. Although social exclusion and loneliness are different concepts, they are strongly correlated [50], and both have been shown to be on the rise in the current context of economic changes -e.g. changes in the employment domain and decreased housing accessibility. On the other hand, individuals who live alone reported higher loneliness levels, regardless of their socioeconomic situation, as consistently shown by previous studies [11, 12, 34], allegedly because they receive lower social and emotional support than their partnered peers [54]. Moreover, it has also been argued that cultural transformations, e.g. more individualistic values [55], or a de-standardization of life trajectories [56], may also feel individuals excluded, potentially leading to a lack of “meaningfulness” -i.e. the capacity to attach value and significance to one’s life-, which has been shown a major predictor of loneliness [57].

This study has several limitations. First, the cross-sectional design does not allow to rule out reverse causality. It could thus be argued that loneliness, which is often intertwined with poor mental health, may exacerbate the negative perception of socioeconomic determinants and not the other way around [58]. Indeed, sensitivity analyses (see Table A3) revealed that poor mental health partly mediated the association. However, most socioeconomic indicators were still significant after accounting for mental health, suggesting an independent association with loneliness levels. Second, the youngest age group includes individuals up to age 34 but individuals in their early twenties may face different socioeconomic challenges than those in their thirties. However, sensitivity analyses with an additional category showed no significant differences, so we opted for a more parsimonious categorization, which in turn allowed comparison with other studies [40]. Third, the data was gathered during the Covid-19 pandemic. While we can hypothesize that the pandemic may have increased loneliness levels and exacerbated the negative impact of socioeconomic determinants, as shown in previous studies [12, 28], it is not possible to isolate the impact of the pandemic and subsequent lockdowns from other elements that occurred along with the pandemic and may have affected loneliness levels, such as increased housing inaccessibility or loss of purchasing power. In any case, more longitudinal research is needed to disentangle the causal direction between loneliness and its socioeconomic correlates.

## Conclusions

This study shows that socioeconomic factors are significantly associated with loneliness levels, beyond individual sociodemographic characteristics. Moreover, the association with socioeconomic factors was somewhat stronger for women, whereas findings showed a nuanced picture regarding age: older workers experienced material deprivation more negatively, while younger individuals’ loneliness was mainly linked to job insecurity and precarity-related factors. This study highlights the importance of identifying relevant socioeconomic variables for effective prevention and intervention programs. A key implication of these findings is that both researchers as well as policy makers should focus more on emerging socioeconomic factors, such as housing conditions or perceived job insecurity, when addressing loneliness in the population, particularly among young people, since traditional assessments (i.e. income, educational level, occupation) fail to capture the complexity of the association between socioeconomic factors and loneliness.

## Electronic supplementary material

Below is the link to the electronic supplementary material.

Supplementary Material 1

## Data Availability

The data that support the findings of this study were provided by the Agència de Salut Pública de Barcelona (ASPB). It can be made available from the corresponding author upon reasonable request and with the permission of the Agència de Salut Pública de Barcelona (ASPB).

## Footnotes

1 For instance, 40% of University Adjunct Professors in Spain earn less than 600€/month: https://www.publico.es/sociedad/cinco-profesores-universitarios-contrato-544-euros-mes.html.

2 The Spanish term is “Expediente de Regulación Temporal de Empleo (ERTO)”.

3 https://ec.europa.eu/eurostat/statistics-explained/index.php?title=EU_statistics_on_income_and_living_conditions_(EU-SILC)_methodology.
