# Supplementary material for: Emerging socioeconomic correlates of loneliness. Evidence from the Barcelona Health Survey 2021
Source: Soc Psychiatry Psychiatr Epidemiol. 2024 Nov 18;60(4):917–29. doi: 10.1007/s00127-024-02789-w (PMC12031919; doi:10.1007/s00127-024-02789-w)
Supplement: Supplementary file 1 — Supplementary Material 1 [file 127_2024_2789_MOESM1_ESM.docx]

**Supplementary File (online resources)**

**Article title:** Emerging socioeconomic correlates of loneliness. Evidence from the Barcelona Health Survey 2021

**Journal Name:** Social Psychiatry and Psychiatric Epidemiology

**Author names:** Lluís Mangot-Sala, Xavier Bartoll-Roca, Esther Sánchez-Ledesma, Mònica Cortés-Albaladejo, Aart C. Liefbroer, Katherine Pérez.

**Corresponding author:** Lluís Mangot-Sala

**Email:** [lluis.mangotsala@ki.se](mailto:lluis.mangotsala@ki.se)

**Affiliations of corresponding author:**

Netherlands Interdisciplinary Demographic Institute (NIDI) – Royal Netherlands Academy of Sciences (KNAW)

Department of Epidemiology, University Medical Center Groningen (UMCG)

Agència de Salut Pública de Barcelona (ASPB), Barcelona, Spain

Unit of Occupational Medicine, Institute of Environmental Medicine, Karolinska Institutet. Stockholm, Sweden.

**Table A1. Correlates of loneliness. Main effects model with imputed values (*n*=3,337).**

|  | Model 1 (n=3,337) | |
| --- | --- | --- |
|  | b | 95% CI |
| Gender (Men) |  |  |
| Women | 0.11 | (-0.01; 0.23) |
| Age (<35) |  |  |
| 35-50 | 0.00 | (-0.17; 0.16) |
| 51-65 | 0.00 | (-0.20; 0.20) |
| >65 | -0.28 | (-0.58; 0.02) |
| Living arrangement (Married) |  |  |
| Alone | 0.99** | (0.80; 1.18) |
| With other (not partner) | 0.43** | (0.26; 0.58) |
| With partner | 0.37** | (0.20; 0.53) |
| Educational Level (University) |  |  |
| Primary (or lower) | 0.07 | (-0.08; 0.22) |
| Secondary | 0.02 | (-0.12; 0.16) |
| Nationality (Spanish) |  |  |
| Other | 0.33** | (0.16; 0.50) |
| Employment status (Employed full-time) |  |  |
| Employed part-time | 0.22* | (0.03; 0.41) |
| Unemployed | 0.17 | (-0.05; 0.38) |
| Homemaker | 0.52** | (0.21; 0.82) |
| Retired | 0.28* | (0.01; 0.55) |
| Disabled | 1.05* | (0.69; 1.41) |
| Job Insecurity (No insecurity) |  |  |
| Job Insecurity | -0.01 | (-0.19; 0.17) |
| Work during Covid (Employed as usual) | -0.10 | (-0.34; 0.14) |
| ERTO (Furloughed temporarily) |  |  |
| Lost job (laid-off) | 0.54** | (0.25; 0.84) |
| Temporary job (No) |  |  |
| Yes | 0.23* | (0.00; 0.46) |
| Freelance (No) |  |  |
| Yes | -0.02 | (-0.27; 0.22) |
| Work without contract (No) |  |  |
| Yes | 0.34 | (-0.13; 0.80) |
| House tenancy (Owner; paid) |  |  |
| Mortgage | -0.04 | (-0.21; 0.12) |
| Tenant | -0.16* | (-0.32; 0.00) |
| Housing Conditions (Good) |  |  |
| Fair | 0.29** | (0.14; 0.43) |
| Poor | 0.51** | (0.25; 0.78) |
| Housing Insecurity (No insecurity) |  |  |
| Insecurity | 0.41* | (0.09; 0.72) |
| Material Poverty (No) |  |  |
| Poverty | 0.34** | (0.17; 0.52) |
| Financial difficulties (No difficulties) |  |  |
| Moderate difficulties | 0.11 | (-0.02; 0.23) |
| Great difficulties | 0.63** | (0.42; 0.84) |

*p-value <0.05; **p-value <0.01

**Table A2.1 Main effects model stratified by gender. Correlates of loneliness AMONG MEN.**

|  | | Model 1 (n=1,459) | |
| --- | --- | --- | --- |
|  | b | | 95% CI |
| Age (<35) |  | |  |
| 35-50 | 0.09 | | (-0.14; 0.32) |
| 51-65 | 0.07 | | (-0.21; 0.36) |
| >65 | 0.07 | | (-0.39; 0.52) |
| Living arrangement (Married) |  | |  |
| Alone | 1.21** | | (0.90; 1.52) |
| Cohabit. with others (not partner) | 0.43** | | (0.18; 0.68) |
| With partner | 0.55** | | (0.32; 0.78) |
| Educational Level (University) |  | |  |
| Primary (or lower) | -0.14 | | (-0.35; 0.07) |
| Secondary | -0.06 | | (-0.25; 0.14) |
| Nationality (Spanish) |  | |  |
| Other | 0.34** | | (0.11; 0.58) |
| Employment status (Employed full-time) |  | |  |
| Employed part-time | 0.29 | | (-0.03; 0.61) |
| Unemployed | 0.57** | | (0.26; 0.89) |
| Homemaker | 3.65** | | (2.24; 5.07) |
| Retired | 0.14 | | (-0.26; 0.54) |
| Disabled | 1.14** | | (0.60; 1.69) |
| Work during Covid (Employed as usual) |  | |  |
| ERTO (Furloughed temporarily) | 0.12 | | (-0.13; 0.37) |
| Lost job (laid-off) | 0.00 | | (-0.44; 0.43) |
| Job Insecurity (No insecurity) |  | |  |
| (High) Insecurity | 0.25 | | (-0.14; 0.63) |
| Temporary job (No) |  | |  |
| Yes | 0.21 | | (-0.12; 0.54) |
| Freelance (No) |  | |  |
| Yes | -0.03 | | (-0.35; 0.29) |
| Work without contract (No) |  | |  |
| Yes | 0.07 | | (-0.64; 0.78) |
| House tenancy (Owner; paid) |  | |  |
| Mortgage | -0.17 | | (-0.39; 0.06) |
| Tenant | -0.15 | | (-0.31; 0.02) |
| Housing Conditions (Good) |  | |  |
| Fair | 0.16 | | (-0.05; 0.37) |
| Poor | 0.45* | | (0.05; 0.84) |
| Housing Insecurity (No insecurity) |  | |  |
| Insecurity | 0.29 | | (-0.19; 0.76) |
| Material Poverty (No) |  | |  |
| Poverty | 0.05 | | (-0.22; 0.33) |
| Financial difficulties (No difficulties) |  | |  |
| Moderate difficulties | 0.15 | | (-0.02; 0.33) |
| Great difficulties | 0.64** | | (0.34; 0.93) |

*p-value <0.05; **p-value <0.01

**Table A2.2 Main effects model stratified by gender. Correlates of loneliness AMONG WOMEN.**

|  | | Model 1 (n=1,620) | |
| --- | --- | --- | --- |
|  | b | | 95% CI |
| Age (<35) |  | |  |
| 35-50 | -0.08 | | (-0.33; 0.17) |
| 51-65 | 0.01 | | (-0.28; 0.31) |
| >65 | -0.37 | | (-0.80; 0.05) |
| Living arrangement (Married) |  | |  |
| Alone | 0.82** | | (0.55; 1.08) |
| Cohabit. with others (not partner) | 0.34** | | (0.12; 0.56) |
| With partner | 0.20 | | (-0.05; 0.44) |
| Educational Level (University) |  | |  |
| Primary (or lower) | 0.08 | | (-0.35; 0.07) |
| Secondary | -0.06 | | (-0.25; 0.14) |
| Nationality (Spanish) |  | |  |
| Other | 0.23 | | (-0.02; 0.49) |
| Employment status (Employed full-time) |  | |  |
| Employed part-time | 0.10 | | (-0.16; 0.36) |
| Unemployed | 0.06 | | (-0.28; 0.40) |
| Homemaker | 0.50** | | (0.14; 0.87) |
| Retired | 0.45* | | (0.06; 0.84) |
| Disabled | 1.00** | | (0.47; 1.53) |
| Work during Covid (Employed as usual) |  | |  |
| ERTO (Furloughed temporarily) | -0.08 | | (-0.36; 0.19) |
| Lost job (laid-off) | 0.01 | | (-0.45; 0.48) |
| Job Insecurity (No insecurity) |  | |  |
| (High) Insecurity | 0.81** | | (0.38; 1.25) |
| Temporary job (No) |  | |  |
| Yes | 0.16 | | (-0.18; 0.50) |
| Freelance (No) |  | |  |
| Yes | -0.03 | | (-0.41; 0.36) |
| Work without contract (No) |  | |  |
| Yes | 0.53 | | (-0.12; 1.18) |
| House tenancy (Owner; paid) |  | |  |
| Mortgage | 0.12 | | (-0.12; 0.36) |
| Tenant | -0.07 | | (-0.31; 0.16) |
| Housing Conditions (Good) |  | |  |
| Fair | 0.37** | | (0.16; 0.59) |
| Poor | 0.7* | | (0.09; 0.84) |
| Housing Insecurity (No insecurity) |  | |  |
| Insecurity | 0.54* | | (0.11; 0.98) |
| Material Poverty (No) |  | |  |
| Poverty | 0.47** | | (0.22; 0.72) |
| Financial difficulties (No difficulties) |  | |  |
| Moderate difficulties | 0.05 | | (-0.13; 0.24) |
| Great difficulties | 0.66** | | (0.35; 0.97) |

*p-value <0.05; **p-value <0.01

**Table A3. Sensitivity Analyses. Main effects model containing only subsample of individuals reporting “good mental health”^a^.**

|  | Model 1 (n=2,267) | |  |
| --- | --- | --- | --- |
|  | b | 95% CI |  |
| Gender (Men) |  |  |  |
| Women | 0.04 | (-0.05; 0.14) |  |
| Age (<35) |  |  |  |
| 35-50 | -0.06 | (-0.20; 0.08) |  |
| 51-65 | 0.02 | (-0.15; 0.19) |  |
| >65 | -0.12 | (-0.37; 0.12) |  |
| Living arrangement (Married) |  |  |  |
| Alone | 0.62** | (0.46; 0.79) |  |
| Cohabit. with others (not partner) | 0.32** | (0.19; 0.46) |  |
| With partner | 0.32** | (0.19; 0.46) |  |
| Educational Level (University) |  |  |  |
| Primary (or lower) | -0.11 | (-0.23; 0.02) |  |
| Secondary | -0.05 | (-0.17; 0.06) |  |
| Nationality (Spanish) |  |  |  |
| Other | 0.28** | (0.14; 0.43) |  |
| Employment status (Employed full-time) |  |  |  |
| Employed part-time | 0.10 | (-0.07; 0.27) |  |
| Unemployed | 0.42** | (0.22; 0.62) |  |
| Homemaker | 0.26* | (0.00; 0.52) |  |
| Retired | 0.23* | (0.01; 0.45) |  |
| Disabled | 0.49* | (0.12; 0.86) |  |
| Job Insecurity (No insecurity) |  |  |  |
| (High) Insecurity | 0.31* | (0.03; 0.59) |  |
| Work during Covid (Employed as usual) |  |  |  |
| ERTO (Furloughed temporarily) | 0.00 | (-0.16; 0.15) |  |
| Lost job (laid-off) | 0.08 | (-0.20; 0.35) |  |
| Temporary job (No) |  |  |  |
| Yes | 0.28** | (0.08; 0.48) |  |
| Freelance (No) |  |  |  |
| Yes | -0.02 | (-0.22; 0.18) |  |
| Work without contract (No) |  |  |  |
| Yes | -0.12 | (-0.51; 0.27) |  |
| House tenancy (Owner; paid) |  |  |  |
| Mortgage | -0.05 | (-0.18; 0.08) |  |
| Tenant | -0.07 | (-0.20; 0.05) |  |
| Housing Conditions (Good) |  |  |  |
| Fair | 0.22** | (0.10; 0.35) |  |
| Poor | 0.47** | (0.22; 0.72) |  |
| Housing Insecurity (No insecurity) |  |  |  |
| Insecurity | -0.04 | (-0.35; 0.27) |  |
| Material Deprivation (No) |  |  |  |
| Deprivation | -0.13 | (-0.29; 0.04) |  |
| Financial difficulties (No difficulties) |  |  |  |
| Moderate difficulties | 0.02 | (-0.07; 0.12) |  |
| Great difficulties | 0.28** | (0.09; 0.47) |  |
| *p-value <0.05; **p-value <0.01. ^a^GHQ-12 score <3. | | |  |

**General Health Questionnaire – GHQ 12 items:**

*“Have you recently…*

- *Been able to concentrate on what you’re doing?*
- *Lost much sleep over worry?*
- *Felt you were playing a useful part in things?*
- *Felt capable of making decisions about things?*
- *Felt constantly under strain?*
- *Felt you couldn’t overcome your difficulties?*
- *Been able to enjoy your normal day-to-day activities?*
- *Been able to face up to your problems?*
- *Been feeling unhappy and depressed?*
- *Been losing confidence in yourself?*
- *Been thinking of yourself as a worthless person?*
- *Been feeling reasonably happy, all things considered?”*

Answers to each question:

- *Not at all (score 0)*
- *No more than usual (score 0)*
- *Rather more than usual (score 1)*
- *Much more than usual (score 1)*
- Total score: 0-12.

Cut-off for “poor mental health”: ≥3.
